# Supplementary material for: Resolving the full spectrum of human genome variation using Linked-Reads
Source: Genome Res. 2019 Apr;29(4):635–45. doi: 10.1101/gr.234443.118 (PMC6442396; doi:10.1101/gr.234443.118)
Supplement: Supplemental Material [file supp_gr.234443.118_Supplemental_longranger-2.2.zip › longranger-2.2.tar.bz2!/lr2.2-public/lib/go/src/code.google.com/vitess/vitess.io/_includes/pagination.html]

{% capture category\_name %}{{ page.categories | first }}{% endcapture %}
{% for post in site.categories.[category\_name] %}
{% if post.url == page.url %}
{% assign post\_index0 = forloop.index0 %}
{% assign post\_index1 = forloop.index %}
{% endif %}
{% endfor %}
{% for post in site.categories.[category\_name] %}
{% if post\_index0 == forloop.index %}{% assign next\_post = post %}{% endif %}
{% if post\_index1 == forloop.index0 %}{% assign prev\_post = post %}{% endif %}
{% endfor %}
{% if prev\_post %}{{ prev\_post.title }}{% endif %}
{% if next\_post %}{{ next\_post.title }}{% endif %}
